# Limited evidence on the effectiveness of interventions to reduce livestock predation by large carnivores

**DOI:** 10.1038/s41598-017-02323-w

**Published:** 2017-05-18

**Authors:** Ann Eklund, José Vicente López-Bao, Mahdieh Tourani, Guillaume Chapron, Jens Frank

**Affiliations:** 1 Grimsö Wildlife Research Station, Department of Ecology, Swedish University of Agricultural Sciences, SE-730 91 Riddarhyttan, Sweden; 2 Research Unit of Biodiversity (UO/CSIC/PA), Oviedo University, Gonzalo Gutiérrez Quirós s/n, 33600 Mieres, Spain; 3 Faculty of Environmental Sciences and Natural Resource Management, Norwegian University of Life Sciences, P.O. Box 5003, 1432 Ås, Norway

## Abstract

Successful coexistence between large carnivores and humans is conditional upon effective mitigation of the impact of these species on humans, such as through livestock depredation. It is therefore essential for conservation practitioners, carnivore managing authorities, or livestock owners to know the effectiveness of interventions intended to reduce livestock predation by large carnivores. We reviewed the scientific literature (1990–2016), searching for evidence of the effectiveness of interventions. We found experimental and quasi-experimental studies were rare within the field, and only 21 studies applied a case-control study design (3.7% of reviewed publications). We used a relative risk ratio to evaluate the studied interventions: changing livestock type, keeping livestock in enclosures, guarding or livestock guarding dogs, predator removal, using shock collars on carnivores, sterilizing carnivores, and using visual or auditory deterrents to frighten carnivores. Although there was a general lack of scientific evidence of the effectiveness of any of these interventions, some interventions reduced the risk of depredation whereas other interventions did not result in reduced depredation. We urge managers and stakeholders to move towards an evidence-based large carnivore management practice and researchers to conduct studies of intervention effectiveness with a randomized case-control design combined with systematic reviewing to evaluate the evidence.

## Introduction

Predation on domestic animals is an important factor influencing the coexistence between large carnivores and humans^1^. In order to mitigate the negative impact of large carnivores on livestock, modern societies (through governments), non-governmental organisations (NGOs), and individuals invest logistical and budgetary efforts (i.e., public and private money) in a large number of preventive measures (hereafter interventions) that are believed to reduce the risk or impact of depredation (i.e., carnivores attacking, injuring, or killing domestic animals). Selecting the correct intervention to implement in each unique case is challenging. Choosing incorrectly can for instance result in multiple negative consequences, such as higher economic costs than expected due to potential livestock losses and the need for additional interventions, or exacerbate conflicts between different stakeholders. The choice of intervention can make the difference between life and death to domestic animals as well as carnivores^1–4^. Choosing the appropriate intervention is also important for establishing trust in carnivore managing authorities. Mistrust in authorities and/or management strategies can create feelings of frustration, anger, or fear. Feelings of this kind may ultimately enhance the negative view of carnivore conservation and management, and undermine coexistence between humans and large carnivores in multi-use landscapes^5–7^. Additionally, negative feelings could be spurred by a lack of reliable information on the expected costs, side-effects, and effectiveness of interventions. Particularly so in cases when livestock losses continue after the implementation of an intervention, or when the cost of the intervention is high compared to the expected cost of depredation.

Authorities, wildlife managers, and owners of domestic animals face a wide variety of potential interventions to protect domestic animals from large carnivores^8–10^. Interventions range from lethal (e.g., culling) to non-lethal methods (e.g., fences), overarching policy goals (e.g., carnivore population caps), interventions funded by authorities or initiated and undertaken by the affected people (e.g., compensation systems and increased guarding, respectively), to information dissemination (e.g., public meetings). Lethal interventions have traditionally been widely utilized and played an important role in the reduction and extirpation of large carnivore populations around the world, including Europe and North America^1^. Lethal interventions currently receive less public support than in the past^11,12^ and their implementation on endangered populations contributes to controversy over their use. Some non-lethal interventions have long histories of use, such as livestock guarding dogs^13^, whereas others are based on new technologies, such as scaring devices or electric fencing^14,15^. Carnivore depredation on livestock has occurred for thousands of years, at least since the expansion of livestock husbandry after domestication ca. 11,000 BP^16^. Due to the long and extensive use of various interventions, it could be expected that the best interventions to reduce the impact of large carnivore depredation are by now well tested and identified. Yet, scientific evaluations of interventions are still surprisingly scarce^17,18^ and, in general, our understanding of their efficacy is based on narrative review^19^.

With this study we aim to move closer to the evidence-based practice and systematic reviewing process that has increased the efficiency of medical interventions^20^, and which is actively increasing the efficiency and trustworthiness of biodiversity conservation^21^. We look for evidence of the outcome of implementing interventions, to assess to what extent interventions reduce the risk and impact of attacks by large carnivore on livestock, i.e. how effective interventions are to prevent depredation. This information is critical for the owners of domestic animals, who need to know if the interventions that they spend time and money on actually prevent the loss of livestock. A true systematic review^22^ is beyond the scope of this paper, but we use a structured methodology based on systematic review procedures. Our aim is to answer the fundamental question “What works?” regarding interventions designed to reduce the risk and impact of large carnivore depredation on domestic animals. The objective is to provide a quantitative assessment of the efficacy of evaluated interventions by exhaustively reviewing empirical studies, without building upon previous reviews^8,17,18^, and using a transparent and replicable methodology. Based on our findings, we discuss what scientific evidence is currently available and where there might be room for improvements in large carnivore management science.

## Results

Our initial literature search returned 27,781 publications, of which 562 were read in full (see Methods for search and screening criteria). Only 21 (3.7%) of the 562 reviewed publications fulfilled our inclusion criteria (see Supplementary Table S1). The limited number of studies selected (mean number of studies by intervention group: 3.2, range 1–6, Supplementary Table S1) meant we were unable to carry out meta-analysis on the effectiveness of interventions. Some publications included more than one study, such as testing different interventions in one setting^23,24^, or one intervention tested on several carnivore species or livestock types^25–29^. Out of the 30 carnivore species considered, the final 21 publications focused on 10 species (see Supplementary Table S1). All publications included at least one study of an intervention that reduced the risk of carnivore attack, whereas five publications (24%) also included studies where interventions had either no effect, or led to an increase in livestock depredation.

### Change livestock type

**Figure 1:**
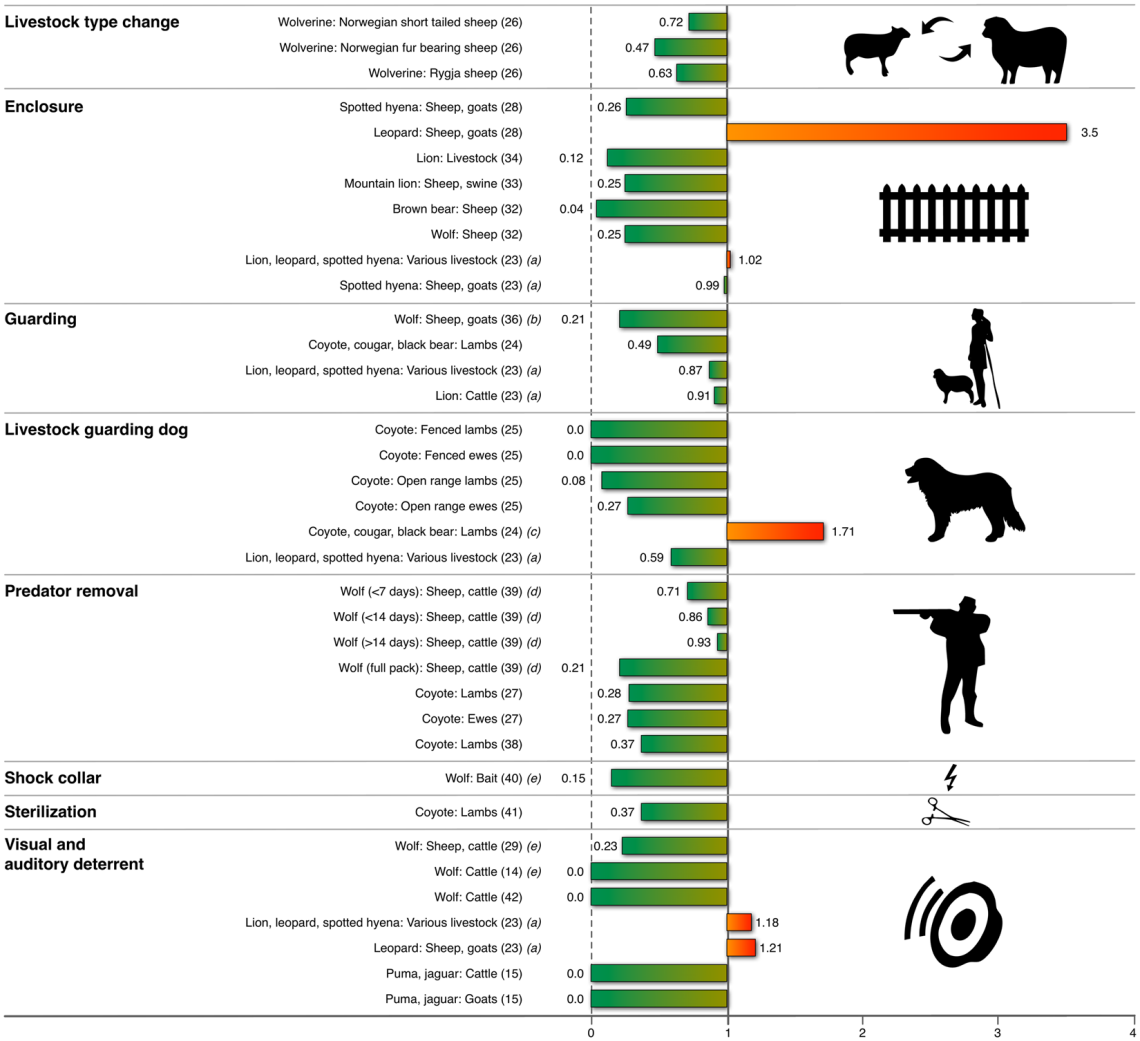
The intervention effectiveness described as relative risk (RR) for each study. RR = 1 suggests no difference in the risk of carnivore attack between treatment and control groups. RR > 1 suggests there is a higher risk of carnivore attack in the treatment group, and the value can be infinitely large. RR < 1 suggests that there is a lower risk of carnivore attack in the treatment group, and the minimum possible value is 0 (no attack in the treatment group). Each row in the figure represents a study or sub-study of an intervention in a certain setting, with the carnivore species and type of livestock described in the figure. Reference to the original publication is written in brackets. For more information of each study please refer to Supplementary Table S1. Additional information for particular studies: (**a**) For Woodroffe *et al*.^23^, odds-ratios were converted to RR using an online Odds Ratio to Risk Ratio calculator^64^. (**b**) Iliopoulos *et al*.^36^ measure severity of the wolf attack once it has occurred. (**c**) Palmer *et al*.^24^ b state that the treatment herd was divided into two bands, and all losses occurred in the band without the livestock guarding dog. (**d**) For Bradley *et al*.^39^ we report hazard ratio HR (1850 days) from the original study. (**e**) Hawley *et al*.^40^, Davidson-Nelson & Gehring^29^, and Lance *et al*.^14^ measure trespass rate into baited areas instead of livestock losses.

One study evaluated the effect of livestock breed on livestock depredation^26^ and found that the heavier breed, *Dala sheep* (focal group), suffered more losses to wolverine (*Gulo gulo*) depredation than did the lighter *Norwegian short-tailed sheep* (relative risk RR_wolverine depredation_ = 0.72), *Norwegian fur-bearing sheep* (RR_wolverine depredation_ = 0.47), and *Rygja sheep* (RR_wolverine depredation_ = 0.63) breeds (Fig. 1). This suggests that the choice of livestock breeds in each particular context (considering also the carnivore species present in the area) can have an effect on losses. *Dala sheep* are heavier than the *Norwegian short-tailed*, the *Norwegian fur-bearing*, and the *Rygja* breeds, with ewe weights of 80–90 kg, 60–70 kg, 60 kg, and 75 kg, respectively^30,31^.

### Enclosure

Six studies focused on the effect of livestock confinement to avoid carnivore depredation. Livestock are often confined during the night, when carnivores are most active. A negative effect on livestock depredation, was found for keeping sheep in night barns in Slovenia^32^ (RR _bear depredation_ = 0.04, RR _wolf depredation_ = 0.25) and also for using night corrals to protect livestock from pumas *(Puma concolor)* in southern Brazil^33^ (RR_puma depredation_ = 0.25, Fig. 1). The most effective enclosure design appears to be context-dependent, and dependent on the carnivore guild, as different species apply different tactics to enter enclosures (e.g. climbing, digging or jumping). A pole construction kept spotted hyenas *(Crocuta crocuta)* out of sheep and goat enclosures better than a bush fence construction^28^ (RR_hyena depredation_ = 0.26), but the enclosure of pole construction left livestock more susceptible to leopard *(Panthera pardus)* depredation than bush fence enclosures^28^ (RR_leopard depredation_ = 3.50). In another study with multiple carnivores, including leopards, the transparency of the enclosure did not have any effect (RR_lion, leopard, hyena depredation_ = 1.02, RR_hyena depredation_ = 0.99, with increasing transparency) on the risk of carnivore depredation^23^. However, fortified or improved enclosures were effective in systems with lions^34^ *(Panthera leo)* (RR_lion depredation_ = 0.12, Fig. 1) and appear to reduce the impact by spotted hyenas^35^ (see Supplementary Table S1).

### Guarding

Three studies fulfilled our requirements for evaluating the effectiveness of guarding. Estimates of RR suggest that herders reduced the severity of wolf *(Canis lupus)* attacks on sheep in Greece^36^ (RR_wolf depredation_ = 0.21, Fig. 1). The risk of coyote (*Canis latrans*), puma, and black bear (*Ursus americanus*) predation was also lower in sheep herds where human herders were present^24^ (RR_coyote, puma, black bear depredation_ = 0.49), and a larger number of men present in a boma had a small negative effect on the depredation risk (RR_lion, leopard, sp. hyena depredation_ = 0.87) by lions, leopards, and spotted hyenas^23^ (Fig. 1).

### Livestock guarding dogs

Five studies met our requirements for evaluating the effectiveness of livestock guarding dogs. Four studies suggest a lower risk of sheep predation where guarding dogs were present^13,23,25,32^. In one study^23^, the dogs were not typical livestock guarding dog breeds, but an increasing number of dogs nevertheless reduced the total risk of carnivore attacks (RR_lion, leopard, sp. hyena depredation_ = 0.59, Fig. 1) likely by alerting people^23^. In four other studies, dogs were guarding breeds that could alert human herders, but may also have been able to deter attacks, or attack the carnivore themselves. Guarding dogs present in sheep herds reduced the risk of depredation of sheep, particularly where herds were fenced (RR_coyote depredation_ = 0 for lambs, RR_coyote depredation_ = 0 for ewes), but also on open range pastures (RR_coyote depredation_ = 0.08 for lambs and RR_coyote depredation_ = 0.27 for ewes, Fig. 1)^25^. One study had increasing depredation losses to coyotes, cougars, and black bears on the herd level (RR_coyote, cougar, black bear depredation_ = 1.7) when the livestock guarding dog was present^24^ (Fig. 1). However, this result was likely due to the insufficient statistical unit (herd) used to measure the effect, as only part of the unit was guarded by a dog and the flock of sheep in which the dog was kept, suffered no losses to carnivores^24^.

### Predator removal

Removal of carnivores can be accomplished through translocation or by various methods of lethal control. Lethal control can target all individuals in an area (i.e., proactive culling)^3^, using methods such as trapping^37^ or aerial shooting^38^. Alternatively, lethal control might target specific individuals within a population that pose a higher threat to livestock than the average individual - i.e. “problem individuals”^27,37^, through selective trapping, poisoning, or hunting. Four studies met our criteria for evaluating the effect of predator removal, all on coyotes and wolves^27,37–39^. Only one considered cases of translocation in their dataset^39^. The largest decrease in risk of livestock depredation (RR_coyote depredation_ = 0.27 for ewe predation and RR_coyote depredation_ = 0.28 for lamb predation^27^) was shown in studies where adult or breeding canids were selectively removed^27,37^ (Fig. 1). A third study found partial wolf pack removals had a smaller negative effect (Hazards Ratio HR_wolf depredation_ = 0.71) on the risk of recurring depredations than full pack removal (HR_wolf depredation_ = 0.21) by lethal control or successful translocation over a period of 1,850 days^39^. An important time aspect of successful removal actions to prevent recurring depredation events was identified: if partial pack removal was accomplished within 7 days there was a slight negative effect on the probability of recurring depredations (HR_wolf depredation_ = 0.71), after 7 days the effect was reduced (HR_wolf depredation_ = 0.86), and 14 days after the first depredation event no effect remained (HR_wolf depredation_ = 0.99), when compared to no wolf removal at all (Fig. 1)^39^. One study of non-selective proactive culling in predefined areas, found that the intervention could still reduce the risk of coyote depredation^38^ (RR_coyote depredation_ = 0.37), although the effect appears smaller than for selective removal of individuals (Fig. 1). To achieve this effect with proactive culling before the grazing season, the total number of coyotes removed in the treatment pastures was more than twice (5.7 ± 1.1) the number removed in the control pastures where coyote removal only occurred during the grazing season (2.0 ± 1.0)^38^.

### Shock collar

Wolves treated with an electric shock whilst approaching a baited site altered their behaviour and reduced their visitation rate to the bait site during the treatment period, in comparison to control wolves that carried a collar without an electric shock devise (RR_wolf trespass_ = 0.15, Fig. 1)^40^. Importantly, conditioning against bait sites was not clearly observed once shocking ceased^40^.

### Sterilization

One study evaluated the effect of sterilization on modifying coyote predatory behaviour^41^. Sterilization of coyote packs eliminates the need for coyotes to provide food for pups. The study found that, in comparison to control coyote packs, the intervention led to a reduction in lamb losses (RR_coyote depredation_ = 0.37, Fig. 1), but it did not eliminate lamb losses entirely in territories where coyote packs were sterilized^41^.

### Visual & Auditory deterrents

Three studies evaluated the effect of fladry (i.e., a rope or line running around an enclosure and from which strips of fabric are suspended) on manipulating carnivore movement in a way that might be expected to reduce livestock depredation. A field trial observed that livestock depredation by wolves ceased in treatment pastures, whilst it continued in control fields (RR_wolf depredation_ = 0, Fig. 1)^42^ and another trial found that wolves did not trespass into fladry pastures whereas they did trespass into control fields (RR_wolf trespass_ = 0.23, Fig. 1)^29^. On the contrary, coyotes trespassed into treatment fields whilst they did not trespass into control fields, suggesting no effect in reducing coyote trespassing^29^. An electrified fladry was tested during a 3-week trial, and wolves did not trespass in treatment fields but did trespass in control fields (RR_wolf trespass_ = 0, Fig. 1)^14^. The same study^14^ detected no effect on livestock losses. Regarding fladry, the timespan during which the intervention can remain effective should be considered. For example, Musiani *et al*.^42^ observed an effect for 60 days, after which wolves trespassed into a treatment pasture and killed livestock again, suggesting that the intervention may have an effect whilst it remains a novelty to the wolves. Other visual deterrents (e.g., hung clothes), combined with auditory deterrents, were found to repel depredation by large neotropical felids^15^. However, visual interventions could potentially also function as an attractant. An evaluation of the effect of the number of scarecrows on an enclosure (thorn-bush boma), to reduce livestock depredations, found that a higher number of scarecrows was associated with a higher number of attacks (RR_lion, leopard, hyena depredation_ = 1.18, RR_leopard depredation_ = 1.21, Fig. 1)^23^.

## Discussion

After reviewing 562 scientific publications addressing livestock depredations by large carnivores from 1990 to 2016, our study reveals a worrying result with substantial implications for large carnivore management: there are not many scientific publications with evidence of effectiveness for any intervention intended to prevent livestock depredation by large carnivores (n = 21). These results are in line with the result of Miller *et al*.^17^ and Treves *et al*.^18^, albeit using a different literature search method and analytical approach, reinforcing the notion that there is very little scientifically published material on the topic, regardless of the literature search methodology. The results presented here could, and should, make us question the presumed effectiveness of widely recognized interventions. Interestingly, some well-known and broadly recommended interventions completely lack scientifically published evaluations, such as carnivore deterring electric fences^43^. But evaluations based on scientifically-sound study designs are also needed to quantify the efficacy of virtually all other interventions.

Nevertheless, the final set of selected studies allows some discussion about the current state of knowledge of intervention effectiveness. Unsurprisingly, the effect of interventions is context dependent and appears to vary with how well the actual problem is targeted. However, identifying the problem locally is rarely easy – the problem could be carnivores of various species, all individuals of a certain species, or even certain individuals within a species^44^. Other carnivore species, or individuals, may be completely unaffected by a specific intervention^28^, or potentially even be attracted to it^23^.

For example, livestock enclosures generally appear to be an effective intervention for protecting livestock from carnivores, but only when the problem species or individual is successfully targeted. In certain cases, the enclosure construction may facilitate entrance by a certain carnivore which may exacerbate livestock losses, likely because livestock are unable to escape the predator. In situations where the carnivore guild is diverse, enclosure constructions may be difficult to design to target multiple species, leading to a reduced total effect of the intervention^23,28^. Nevertheless, where the problem species/individuals are known and can be targeted with suitable enclosure construction, this intervention has great potential for protecting livestock, similarly if the intervention is applied during night-time and the targeted carnivores are mostly nocturnal^32,33^. Enclosures can likely be improved to exclude multiple carnivores if their biology and behaviour is considered during construction.

Livestock guarding, either by humans or by livestock guarding dogs have been used to protect livestock from large carnivores for millennia^45^. These types of interventions appear to be effective measures for reducing livestock losses (Fig. 1). However, there are factors to take into account when implementing these interventions. For example, there are investments (e.g. acquisition of guarding dogs), running costs (e.g. salaries for shepherds or food for guarding dogs), and - in the case of guarding dogs - dog handling legislation associated with their use. Therefore, operators will need to calculate if costs are covered by a reduction of livestock losses for their system, or if there are other benefits that make up the difference. The effect of the livestock guarding dogs appears greater when lambs were fenced in comparison to open range husbandry^25^. Thus, it seems that this intervention could work well in areas where the likelihood of a carnivore attack is high, and where livestock are confined in a way that allows a dog to supervise the flock without straying, particularly at night.

In the past, large-scale predator removal programmes, supported by carnivore eradication policies, brought many carnivore species to regional or national extinction, at which point livestock depredation would cease. In this regard, complete carnivore removal could be considered effective at eliminating livestock depredation. However, carnivores are now more highly valued by society and most large carnivore populations currently benefit from some levels of legal protection that precludes their unregulated killing. For example, the European Habitats Directive 92/43/EEC permits derogating to the strict protection regime for bears, lynx, wolves, and wolverines only to cases where there is no satisfactory alternative to prevent serious damage, in particular to livestock (Article 16.1)^46^. Carnivore removal remains a controversial intervention, and lethal interventions are becoming less popular in society. Nevertheless, where no satisfactory alternative is found to prevent damage on livestock, some predator removal is still used as an intervention. Unselective predator removal may reduce livestock losses^38^ unless the removed individuals are instantly replaced by new individuals, or represent a part of the population that does not kill livestock. In this case predator removal may be completely inefficient^37,39^. On the contrary, where problem individuals can be identified and specifically targeted, this intervention may have greater potential^27,37^. A recent publication showed that the removal of entire wolf packs reduced the occurrence of recurring depredations, whereas partial pack removal was only marginally more efficient than no removal^39^. Although the social status of removed individuals is not reported, a full pack removal - where all individuals are essentially removed - would include potential “problem individuals”, whereas they could have been missed in a partial pack removal. The timing of predator removal can also influence the effectiveness of the intervention^39,47^, either because most recurring depredation events occur within a limited time, or due to different movement patterns in the carnivore population. If culling occurs before the dispersal season, it may be more likely that new depredating individuals reclaim an area, than if culling is made after the dispersal season^48,49^. Additionally, the population and social structure of the target species can influence the effectiveness and suitability of lethal interventions. For instance, predator removal from source populations may be more effective in reducing livestock depredation compared to removals in sink populations (removed individuals in sink populations may be replaced by new individuals quicker). However, removal of predators from source populations may also be more detrimental to the carnivore populations^50^. Controversy exists about the potential side effects of lethal control, such as disruption in carnivore social structure that could lead to increased immigration causing further livestock depredation^47,51–53^.

Visual and auditory deterrents represent ways of non-lethal control that propose alternatives to lethal control. Fladry is a historically utilized visual deterrent that has received some attention in research. Cloth flags hung from a rope were used historically to steer wolves during hunts, and now fladry is used to repel wolves from areas with livestock. Sample sizes are limited, but studies indicate that fladry can manipulate wolf movement in a way that is expected^14,29^, or observed^42^, to reduce livestock losses. Electrical fladry may have additional repelling properties, but is costlier and needs more maintenance to be fully functional^14^. The need for maintenance also means that fladry lines are frequented by humans^29,42^, likely adding additional human scent and presence to the area. An interesting prospect for future studies would be to disentangle this effect from the effect of the cloth line itself. Over time, as the fladry becomes a familiar feature in the wolves’ environment^42^, or due to mechanical failures, e.g., wear and tear, or entanglement, the effectiveness of the intervention decrease^54^. The application time of an intervention is therefore important to address. Indeed, some of the reviewed studies were of limited duration^14,40,41^, and many more lack a clear description of the timeframe during which interventions were implemented. Future studies would benefit from considering closely the study length, to better evaluate the length of time that intervention implementation is expected to reduce losses.

Several interventions – *change livestock type*, *shock collar*, and *sterilization* - have only been properly evaluated in one single study. Generalizing the effects of these interventions on livestock losses is, therefore, practically impossible. Choosing a type of livestock species or breed that is less prone to predation may, in some situations, reduce livestock losses^26^. Nevertheless, unless we gain knowledge about what types of livestock are more resilient to particular predators, livestock owners are at risk of choosing a type of livestock that is actually more, or equally, prone to depredation. In this case the intervention could be useless or even counterproductive. It may thus be better to refrain from using an expensive or effort intense intervention like changing livestock type, until more evaluations are made. Meanwhile, practitioners can choose among those interventions for which there is at least some evidence available.

Policy makers and practitioners should also give thorough consideration to the feasibility of interventions, before advocating its use. For instance, shock collars can potentially train wolves to avoid livestock herds^40^, but it may not be financially or logistically feasible to collar all carnivores in an area, and uncollared individuals could still kill livestock. In such situations, managers and livestock owners are likely better off considering other interventions. Likewise, sterilizing reproducing animals may reduce livestock losses^41^, but this intervention may be very expensive, ethically unacceptable, and counterproductive in achieving conservation goals.

We suggest that feasible interventions, i. e. low-cost interventions that build on existing technology and can be easily implemented in multiple contexts, should be prioritized for scientific evaluation. This could be achieved by testing the effects of different versions or applications of already existing interventions such as guarding or enclosures. Scientific evaluations of intervention effectiveness are needed to improve, and increase the trustworthiness of, large carnivore management. Whilst carnivore management relies heavily on interventions to protect livestock from carnivore attack, the scientific evidence supporting intervention effectiveness is sparse, highlighting the need for further investigations to provide scientifically-sound information on what interventions work. It is questionable if the contemporary scientific evidence is even solid enough to allow generalized assumptions about the effectiveness of the presented interventions, not least as the few studies which have attempted to evaluate intervention effectiveness deal with different systems and carnivore species. As scientific evaluations are generally lacking, it is likely that the choice of interventions is often based on expert opinion, rather than evidence^2,8,43^. The time and money spent on interventions could likely be used more efficiently, if there was evidence available to guide management choices.

While in a long term perspective it would be beneficial to know the effect of interventions before investing in them, the short term goals for farmers, managers and researchers may not be in favour for these kinds of studies being conducted in the near future. Attempts to increase the involvement of these actors, contributing together to evidence-based approaches, may be one way to alter the odds in a favourable direction. We are not suggesting that farmers or managers should do nothing until evidence is available, but merely encourage these actors to promote collaborative approaches, and work together in order to increase the proportion of studies aiming to quantify the effect of interventions. Within systems that aim for adaptive management of large carnivores, the use of interventions should preferably be applied in a way that allows scientific evaluation of intervention effectiveness^55^. Special attention should focus to match comparable treatment and control samples. A limitation in this regard has been that interventions often are allocated to areas or situations where the risk of predation is higher whilst the controls are allocated to areas or situations where the risk is lower, thus the pair does not match.

Although in this study we have reviewed the existing scientific evidence using the broad scope of the Zoological Record database, we are unable to answer the question “What works ? ” with regards to intervention effectiveness to prevent carnivore attacks on livestock. This inability is not caused by flaws in our methodology, but by a lack of robust studies able to identify reliable answers, which some have suggested also applies to other large carnivore questions^55^. It is possible that some studies were overseen in this literature review as we limited our search to one database and excluded grey literature and literature published in languages other than English. We chose to exclude grey literature because the scientific contribution was an important focus of this review, and we believe that this exclusion criterion did not eliminate a large number of studies. While including only peer-reviewed papers we may be at risk of some publication bias, as it may be harder to publish studies when no effect of interventions was found, peer review provides a quality control for the included studies, reducing the risk of incorrect conclusions in our review. We did not set any geographical limitations to our literature search, but the 21 studies fulfilling our criteria were limited to the African (n = 4), European (n = 3), North American (n = 13), and South American (n = 1) continents, thereby limiting the study species to large carnivores present in these ranges (see Supplementary Table S1).

Ultimately, the field needs to move toward an evidence-based practice informed by regular gold standard systematic reviews – with published peer-reviewed protocols and replicable searches for scientific, as well as grey, literature in multiple relevant databases and websites - to turn the management of large carnivores into a cost effective and trustworthy practice^22^. We also suggest that future large carnivore management adopts the basic principles of an adaptive approach^56^ and plan interventions to allow evaluations of effect and causality. We hope researchers embrace the challenge to improve study designs and move towards solid evaluations of management interventions.

We fully acknowledge the difficulties facing research projects studying large carnivores and livestock and that it is far from always possible to perform randomized controlled trials. Nevertheless, we suggest the field of large carnivore management follows the lead of medical sciences and conservation practices, and aim to produce evidence of the highest possible quality^57^. If we continue to do research in the way we have so far been doing, many more papers will be published in the coming years, but likely providing very little reliable knowledge on the effectiveness of interventions that cost farmers and tax payers vast amounts of money, as well as the lives of livestock and carnivores around the globe.

## Methods

### Literature review

To be included in this literature review, studies had to *i)* be written in English and published in a peer-reviewed scientific journal; *ii)* include an empirical study of wild (i.e., not captive) carnivores; *iii)* include a quantitative evaluation of interventions to prevent/reduce depredation of livestock (excluding apiaries); *iv)* include a description of the methods used to implement the intervention (treatment) and of a study design sufficient for replication; and *v)* include a matched control to which the treatment was compared.

We compiled a database from the *Zoological Record* (http://wokinfo.com/products_tools/specialized/zr/) containing publications between 1 January, 1990 and 16 June, 2016. The search was made with the subject descriptors “*Carnivora OR Canidae OR Felidae OR Hyaenidae OR Mustelidae OR Procyonidae OR Ursidae OR Viverridae OR Viverridae”*. In total, we retrieved 48,894 titles. The titles and abstracts of these publications were imported to EndNote X7.0.2 (Thomson Reuters, New York, United States) and screened by the following search string: “*depredation OR stock OR poultry OR damage OR mitigation OR conflict OR control OR cull OR cow OR bull OR calf OR calves OR chicken OR hen OR ewe OR lamb OR pet OR cat OR hound OR pony OR ponies OR mule OR reindeer OR llama OR yak OR buffalo OR livestock OR cattle OR sheep OR goat OR horse OR pig OR dog OR attack OR camel OR donkey”*. With this screening, we were left with 27,781 publications.

We manually screened the remaining publications to identify studies written in English that dealt with depredation of domestic animals (livestock and pets) by terrestrial mammalian large carnivores. Large carnivores were defined as species with an average body mass of >15 kg. We included studies of the 28 species listed by Ripple *et al*.^58^ as well as coyotes and wolverines. These species were included because their body size can be larger than 15 kg^59,60^ and conflicts associated with livestock depredation occur in different parts of their worldwide range^61,62^. After the first manual screening, two co-authors (AE and MT) read the 562 remaining publications in full. During the full text screening, we identified whether studies were correlational, quasi-experimental, or experimental. Experimental studies should include a randomized case-control study design, whereas studies were considered quasi-experimental if a case-control study design was used, but not assigned randomly. We also identified which studies included a quantitative measure of the effectiveness of an intervention. More precisely, the effectiveness of the intervention should be measured as the number of livestock killed, the number of attacks on livestock units, or the ability of the intervention to manipulate carnivore behaviour/movement in a way that is expected to reduce exposure of livestock to carnivore predation, e.g., by preventing trespasses into a baited exclusion area. We refer to all these instances as depredation events. A panel of two additional co-authors (JF and JVLB) additionally read all studies where intervention effectiveness was quantitatively evaluated, but where some uncertainty remained about the classification (correlational, quasi-experimental, or experimental), after which we collectively determined the classification. Only publications that included a quantitative measure of the effectiveness of an intervention and had an experimental or quasi-experimental study design, were included (i.e., all correlational studies were excluded). At this stage, we also removed one publication that had not gone through a scientific peer-review process. In the end, 21 scientific papers remained, describing 34 evaluations of intervention effectiveness.

### Grouping interventions

We defined eight intervention groups: Change of livestock type, Enclosure, Guarding, Livestock guarding dog, Predator removal, Shock collar, Sterilization of carnivore, and Visual & Auditory deterrents. We aimed to group the interventions as specifically as possible to aid in the interpretation of between-intervention effectiveness. An even more specific grouping (e.g., trapping, shooting, poisoning, translocation instead of “predator removal”) might have revealed more detailed information of between-intervention effectiveness, but was not possible due to the low number of studies that met our criteria (see Results). A broader categorization of interventions would involve a risk that the effect of one specific intervention could be masked by the effect of other interventions within the same category. Two co-authors (AE and JF) qualitatively summarized the results of the studies within each intervention group in the synopses presented in the Results section.

### Data analyses

To allow a quantitative comparison of effectiveness between interventions, we calculated the relative risk (or risk ratio, RR) for carnivore depredation in treatment vs. control groups for each study^63^. For studies that measured the effect of an intervention in preventing trespasses, we used the number of incursions instead of the number of depredated animals. With the RR, we obtained an estimate for the effectiveness of the intervention (treatment) in comparison to using no intervention (control) in the same setting^63^. The relative risk is defined as the ratio between the probability of depredation by large carnivores in the treatment group and the probability of livestock depredation by large carnivores in the control group:

1 $${\rm{Relative}}\,\,{\rm{Risk}}({\rm{RR}})=\frac{a/(a+b)}{c/(c+d)}$$

where *a* is the number of depredated animals/units in the treatment group, *b* is the number of unharmed animals/units in the treatment group, *c* is the number of depredated animals/units in the control group, and *d* is the number of unharmed animals/units in the control group. In cases where there is no difference in the risk of depredation between the treatment and the control group, the relative risk is 1. When RR > 1, the risk of depredation is more likely to occur in the treatment group (with larger values of RR indicating a counter-productive intervention), and for RR < 1 depredation risk is higher in the control group (with values of RR indicating a greater intervention effectiveness as they get close to 0).

We took slightly different approaches to calculate RR, depending on the statistical units or reported statistics in the various studies. When possible, we used the mean number of animals in treatment and control herds, as reported in the original studies (n = 1). In studies where true numbers of herd size and depredation loss were reported for several herds separately, we first calculated the average herd size and the average numbers of depredated animals in treatment and control groups, and used these means to calculate RR (n = 11). For studies that reported the number of livestock units that suffered depredation and the number of livestock units that were unharmed, we used the number of livestock units for our calculation of RR (n = 2). In two cases, odds-ratios (OR) and hazards-ratios (HR) were reported in the original papers. Odds-ratios were converted to RR using an online odds ratio to risk ratio calculator^64^, whereas we report the HR. For five papers that did not report herd sizes, we contacted the corresponding authors by email with a request for that data. All authors kindly responded to the request and we were provided data from two papers^32,36^. All studies considered in this review are included in Supplementary Table S1, and all studies with calculated/converted RR (n = 17) and reported HR (n = 1) are presented in Fig. 1.

## Electronic supplementary material

Table S1
