# Author Correction: Limited evidence on the effectiveness of interventions to reduce livestock predation by large carnivores

**DOI:** 10.1038/s41598-018-23581-2

**Published:** 2018-04-05

**Authors:** Ann Eklund, José Vicente López-Bao, Mahdieh Tourani, Guillaume Chapron, Jens Frank

**Affiliations:** 1 Grimsö Wildlife Research Station, Department of Ecology, Swedish University of Agricultural Sciences, SE-730 91 Riddarhyttan, Sweden; 2 Research Unit of Biodiversity (UO/CSIC/PA), Oviedo University, Gonzalo Gutiérrez Quirós s/n, 33600 Mieres, Spain; 3 Faculty of Environmental Sciences and Natural Resource Management, Norwegian University of Life Sciences, P.O. Box 5003, 1432 Ås, Norway

Correction to: *Scientific Reports* 10.1038/s41598-017-02323-w, published online 18 May 2017

This Article contains a typographical error in the Results section under subheading ‘Enclosure’.

“A negative effect on livestock depredation, was found for keeping sheep in night barns in Slovenia^32^ (RR _bear depredation_ = 0.04, RR _wolf depredation_ = 0.25) and also for using night corrals to protect livestock from pumas *(Puma concolor)* in southern Brazil^33^ (RR_puma depredation_ = 0.25, Fig. 1).”

should read:

“A negative effect on livestock depredation, was found for keeping sheep in night barns in Slovakia^32^ (RR _bear depredation_ = 0.04, RR _wolf depredation_ = 0.25) and also for using night corrals to protect livestock from pumas *(Puma concolor)* in southern Brazil^33^ (RR_puma depredation_ = 0.25, Fig. 1).”

