# Supplementary material for: Limited evidence on the effectiveness of interventions to reduce livestock predation by large carnivores
Source: Sci Rep. 2017 May 18;7:2097. doi: 10.1038/s41598-017-02323-w (PMC5437004; doi:10.1038/s41598-017-02323-w)
Supplement: Supplementary file 1 — Table S1 [file 41598_2017_2323_MOESM1_ESM.pdf]

Limited evidence on the effectiveness of interventions to reduce livestock predation by large carnivores

Ann Eklund*[1], José Vicente López-Bao[2], Mahdieh Tourani[1,3], Guillaume Chapron[1], Jens Frank[1]

[1] Grimsö Wildlife Research Station, Department of Ecology, Swedish University of Agricultural Sciences, SE-730 91, Riddarhyttan, Sweden

[2] Research Unit of Biodiversity (UO/CSIC/PA), Oviedo University, Gonzalo Gutiérrez Quirós s/n, 33600, Mieres, Spain

[3] Faculty of Environmental Sciences and Natural Resource Management, Norwegian University of Life Sciences, P.O. Box 5003, 1432 Ås, Norway

Corresponding author:

Ann Eklund, phone: +46 (0)581 69 73 40, email: ann.eklund@slu.se

Table S1. Distribution of all studies identified as applying a quasi-experimental or experimental design, among intervention groups.

Intervention group	Study	Intervention type	Carnivore species	Livestock	Sample size	Duration of study
Change livestock	Landa et al.[26]	Replace Dala sheep breed (70-80 kg) with<br>a) Norwegian short tail breed (65-70kg)<br>b) Norwegian fur bearing breed (60 kg)<br>c) Rygja breed (75-80 kg)	Wolverine (*Gulo gulo*)	a) Lambs<br>b) Lambs<br>c) Lambs	One area,<br>3877 control (Dala) lambs<br>a) 2986 treatment lambs<br>b) 681 treatment lambs<br>c) 428 treatment lambs	3 years
Enclosure	Bauer et al. [35]	Improved enclosures (clay construction)	Lion (*Panthera leo*) and spotted hyena (*Crocuta crocuta*)	Cattle and shoats	2 areas with treatment and control enclosures	2 years
	Kolowski & Holekamp [28]	Use of pole boma vs. bush boma	a) Spotted hyena (*Crocuta crocuta*)<br>b) Leopard (*Panthera pardus*)	Sheep and goats	40 treatment boma<br>44 control boma	14 months

Lichtenfeld et al. [34]	Fortified boma (living wall)	Lion (*Panthera leo*)	Livestock	62 treatment boma<br>84 control boma	9296 boma months/<br>10 years
Mazzolli et al.[33]	Night corrals	Mountain lion (*Puma concolor*)	Sheep and swine	5 treatment farms<br>19 control farms	3 years
Rigg et al. [32]	Night barn (always or sometimes) with pasture as control	a) Brown bear (*Ursus arctos*)<br>b) Wolf (*Canis lupus*)	Sheep	52 treatment farms<br>106 control farms	3 years
Woodroffe et al. [23]	Transparency of boma wall	a) Lion (*Panthera leo*), leopard (*Panthera pardus*), and spotted hyena (*Crocuta crocuta*)	a) Cattle, sheep, goats, camels, and donkeys	a) 178 matched bomas (treatment + control)	4.5 years

			b) Spotted hyena (*Crocuta crocuta*)	b) Sheep and goats	b) 95 matched bomas (treatment + control)	
Guarding	Iliopoulos et al. [36]	Herders vs. non-predator proof fence (damage claims for attacked herds)	Wolf (*Canis lupus*)	Sheep and goats	181 treatment<br>7 control	21 months
	Palmer et al. [24]	Herders	Coyote (*Canis latrans*), cougar (*Puma concolor*), and black bear (*Ursus americanus*)	Lambs	5 treatment herds<br>4 control herds<br>(5 herds, 1-2 years)	4 months
	Woodroffe et al.[23]	Number of men	a) Lion (*Panthera leo*), leopard (*Panthera pardus*), and spotted hyena (*Crocuta crocuta*)<br>b) Lion (*Panthera leo*)	a) Cattle, sheep, goats, camels, and donkeys<br>b) Cattle	a) 178 matched bomas (treatment + control)<br>b) 31 matched bomas (treatment + control)	4.5 years

Livestock guarding dog	Andelt [25]	Livestock guarding dog in<br>a) fenced pastures<br><br>b) open range pastures	Coyote (*Canis latrans*)	 a1) Lambs<br>a2) Ewes<br>b1) Lambs<br>b2) Ewes	 a1) 6 treatment, 73 control<br>a2) 7 treatment,78 control<br>b1) 10 treatment, 20 control<br>b2) 10 treatment, 20 control	1 year
	Gehring et al. [13]	Livestock guarding dog	Coyote (*Canis latrans*)	Cattle	6 treatment farms,<br>3 control farms	3 years
	Palmer et al. [24]	Livestock guarding dog	Coyote (*Canis latrans*), cougar (*Puma concolor*), and black bear (*Ursus americanus*)	Lambs	1 treatment herd<br>8 control herds<br>(5 herds, 1-2 years)	4 months
	Rigg et al. [32]	Livestock guarding dog	Wolf (*Canis lupus*) and brown bear (*Ursus arctos*)	Sheep	14 treatment flocks<br>45 control flocks	4 years

	Woodroffe et al. [23]	Domestic dog present in boma	Lion (*Panthera leo*), leopard (*Panthera pardus*), and spotted hyena (*Crocuta crocuta*)	Cattle, sheep, goats, camels, and donkeys	178 matched bomas (treatment + control)	4.5 years
Predator removal	Bradley et al. [39]	Trapping and euthanizing, ground shooting, aerial gunning, translocation in response to attack. Partial- or full pack removal (with time after first attack).<br>a) partial <7 days<br>b) partial <14 days<br>c) partial >14 days<br>d) full pack removal	Wolf (*Canis lupus*)	Sheep, cattle, and other	326 partial removal<br>48 full pack removal<br>593 control	1850 days
	Blejwas et al. [27]	Removal of breeding pair (toxin or shooting)	Coyote (*Canis latrans*)	a) Lambs<br>b) Ewes	a) 14 territories (after + before)<br>b) 20 territories (after + before)	2.8 years

	Harper et al. [37]	a) Trapping of any individual<br>b) Trapping of adult male	Wolf (*Canis lupus*)	Cattle, turkey, and sheep	923 verified depredations	20 years
	Wagner & Conover [38]	Aerial hunting before grazing season	Coyote (*Canis latrans*)	Lambs	21 paired pastures (treatment + control)	3-6 months
Shock collar	Hawley et al. [40]	Shock collar	Wolf (*Canis lupus*)	Bait	5 treatment wolves<br>5 control wolves	28 days
Sterilization	Bromley & Gese [41]	Sterilization of carnivore	Coyote (*Canis latrans*)	Lambs	4 treatment packs<br>8 control packs (1999)	5-23 days
Visual/ Auditory deterrent	Davidson-Nelson & Gehring [29]	Fladry	a) Wolf (*Canis lupus*)<br>b) Coyote (*Canis latrans*)	Sheep and cattle	4 treatment farms<br>4 control farms	75 days

Lance et al. [14]	Electrical fladry	Wolf (*Canis lupus*)	Cattle	6 treatment pastures<br>6 control pastures	Treatment:<br>20 days,<br>control:<br>29 days
Musiani et al. [42]	Fladry	Wolf (*Canis lupus*)	Cattle and bait	2 pastures<br>(treatment + before)	60 days
Woodroffe et al. [23]	Number of scarecrows	a) Lion (*Panthera leo*), leopard (*Panthera pardus*), and spotted hyena (*Crocuta crocuta*)<br><br>b) Leopard (*Panthera pardus*)	a) Cattle, sheep, goats, camels, and donkeys<br><br>b) Sheep and goats	a) 178 matched bomas (treatment + control)<br><br>b) 35 matched bomas (treatment + control)	4.5 years

Zarco-González & Monroy-Vilchis [15]	Auditory and visual deterrents	Puma (*Puma concolor*), and jaguar (*Panthera onca*)	a) Cattle<br>b) Goats	2 treatment farms<br>1 control farm	2 months
